# Supplementary material for: The causal relationship between the human gut microbiota and pyogenic arthritis: a Mendelian randomization study
Source: Front Cell Infect Microbiol. 2024 Nov 26;14:1452480. doi: 10.3389/fcimb.2024.1452480 (PMC11629706; doi:10.3389/fcimb.2024.1452480)
Supplement: Supplementary file 1 [file DataSheet1.zip › mendelian test/ebi-a-GCST90027557.csv_leave_one_out.pdf]

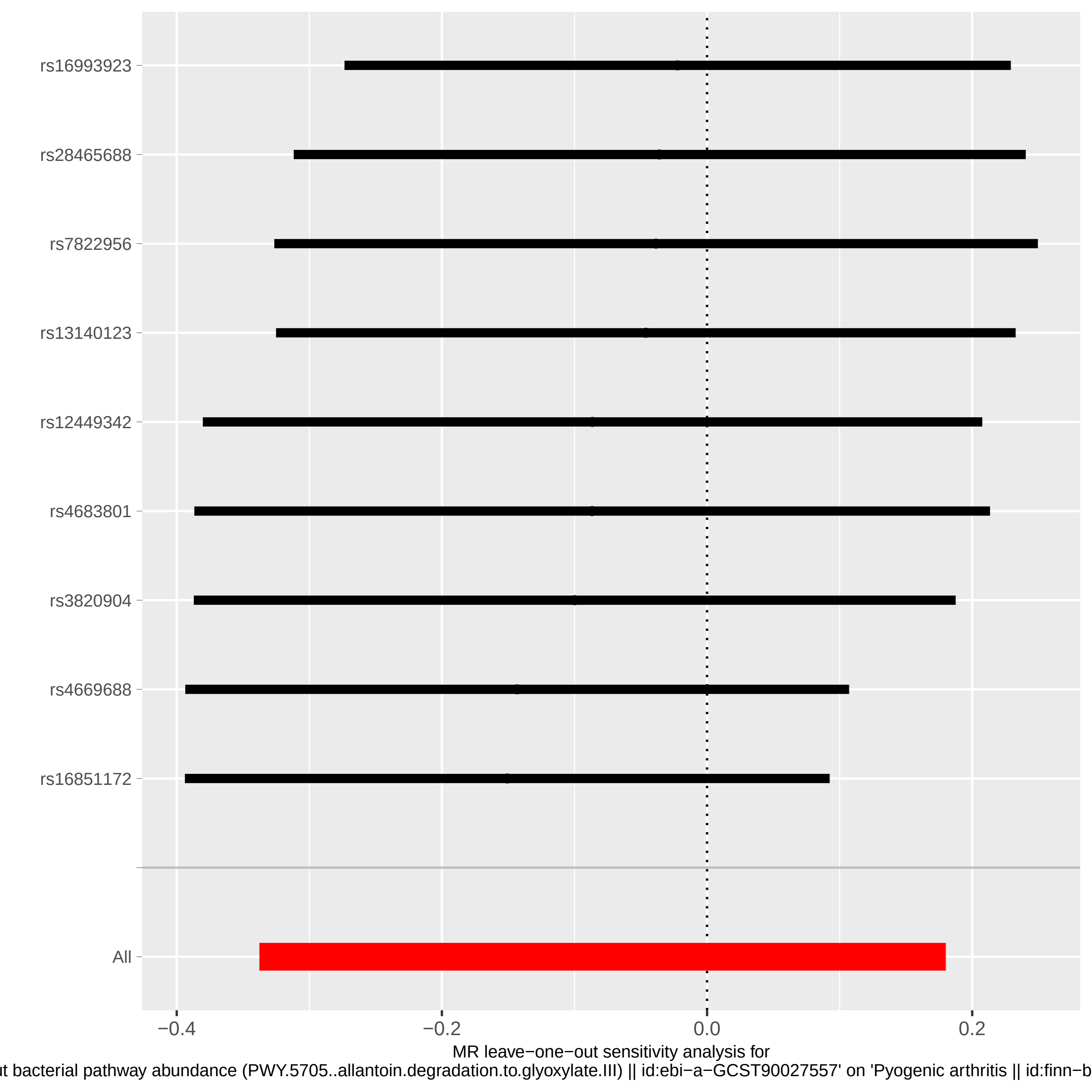

rs16993923
rs28465688
rs7822956
rs13140123
rs12449342
rs4683801
rs3820904
rs4669688
rs16851172
All
−0.4
−0.2
0.0
0.2
MR leave−one−out sensitivity analysis for
ut bacterial pathway abundance (PWY.5705..allantoin.degradation.to.glyoxylate.III) || id:ebi−a−GCST90027557' on 'Pyogenic arthritis || id:finn−b
